# FACTORS ASSOCIATED WITH UNMET HEALTHCARE NEEDS IN OLDER ASIAN AMERICANS

**DOI:** 10.1093/geroni/igz038.120

**Published:** 2019-11-08

**Authors:** Hyunwoo Yoon, Hyunwoo Yoon, Yuri Jang, Kwangyul Choi

**Affiliations:** 1 Texas State University, San Marcos, Texas, United States; 2 School of Social work, Texas state university, San Marcos, Texas, United States; 3 USC Suzanne Dworak-Peck School of Social Work University of Southern California, Los Angeles, California, United States; 4 aculty of Environmental Design, University of Calgary, Calgary, Alberta, Canada

## Abstract

Older Asian Americans are the fast-growing but understudied population in health disparities research. Using a sample that reflects cultural/linguistic diversity, the present study explored general (health insurance, usual place for care, income) and immigrant-specific (nativity, length of stay in the US, English proficiency, acculturation) risk factors of unmet healthcare needs in older Asian Americans. Data were drawn from the Asian American Quality of Life survey (N = 533). With the inclusion of a considerable number of non-English-speaking individuals, the present sample presented a high rate of unmet healthcare needs. Those with a shorter stay in the US, limited English proficiency, and lower level of acculturation had increased odds of having unmet healthcare needs than their counterparts after controlling for background characteristics. Challenging the myth of model minority, findings highlight the importance of immigrant-specific factors in identifying risk groups of unmet healthcare needs and demonstrate vulnerability in access to healthcare.

